# Pyroptotic macrophages induce disruption of glutamate metabolism in periodontal ligament stem cells contributing to their compromised osteogenic potential

**DOI:** 10.1111/cpr.13663

**Published:** 2024-05-27

**Authors:** Li‐Juan Sun, Hong‐Lei Qu, Xiao‐Tao He, Bei‐Min Tian, Rui‐Xin Wu, Yuan Yin, Jie‐Kang Zou, Hai‐Hua Sun, Xuan Li, Fa‐Ming Chen

**Affiliations:** ^1^ State Key Laboratory of Oral & Maxillofacial Reconstruction and Regeneration, National Clinical Research Center for Oral Diseases, Shaanxi International Joint Research Center for Oral Diseases, Department of Periodontology, School of Stomatology The Fourth Military Medical University Xi'an China; ^2^ State Key Laboratory of Oral & Maxillofacial Reconstruction and Regeneration, National Clinical Research Center for Oral Diseases, Shaanxi International Joint Research Center for Oral Diseases, Department of General Dentistry and Emergency, School of Stomatology The Fourth Military Medical University Xi'an China

## Abstract

Macrophage pyroptosis is of key importance to host defence against pathogen infections and may participate in the progression and recovery of periodontitis. However, the role of pyroptotic macrophages in regulating periodontal ligament stem cells (PDLSCs), the main cell source for periodontium renewal, remains unclear. First, we found that macrophage pyroptosis were enriched in gingiva tissues from periodontitis patients compared with those of healthy people through immunofluorescence. Then the effects of pyroptotic macrophages on the PDLSC osteogenic differentiation were investigated in a conditioned medium (CM)‐based coculture system in vitro. CM derived from pyroptotic macrophages inhibited the osteogenic differentiation‐related gene and protein levels, ALP activity and mineralized nodule formation of PDLSCs. The osteogenic inhibition of CM was alleviated when pyroptosis was inhibited by VX765. Further, untargeted metabolomics showed that glutamate limitation may be the underlying mechanism. However, exogenous glutamate supplementation aggravated the CM‐inhibited osteogenic differentiation of PDLSCs. Moreover, CM increased extracellular glutamate and decreased intracellular glutamate levels of PDLSCs, and enhanced the gene and protein expression levels of system x_c_^−^ (a cystine/glutamate antiporter). After adding cystine to CM‐based incubation, the compromised osteogenic potency of PDLSCs was rescued. Our data suggest that macrophage pyroptosis is related to the inflammatory lesions of periodontitis. Either pharmacological inhibition of macrophage pyroptosis or nutritional supplements to PDLSCs, can rescue the compromised osteogenic potency caused by pyroptotic macrophages.

## Graphical abstract

This study revealed that glutamate metabolism plays an important role in the macrophage pyroptosis‐compromised periodontal ligament stem cell osteogenic differentiation. In contrast to previous strategies to change the stem cell fates, such as gene therapy, supplementation with nutrients such as cystine can also improve the performance of stem cells in inflammatory microenvironment.

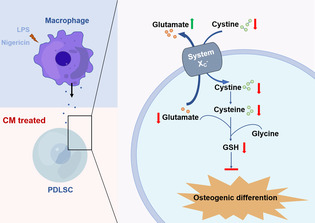

## 1 INTRODUCTION

Periodontitis is an inflammatory disease triggered by bacterial microorganisms and is characterized by the destruction of tooth‐supporting apparatus.^1^ Untreated periodontitis is a prime reason of tooth loss in adults and is associated with multiple systemic diseases.^2^, ^3^, ^4^ Due to the multifaceted properties of stem cells, such as self‐renewal, immunomodulation, multilineage differentiation, and antibacterial effects, periodontal ligament stem cells (PDLSC)‐based therapy is becoming an attractive approach for regenerative medicine.^5^, ^6^, ^7^ Nevertheless, despite the proven efficacy by numerous preclinical studies,^8^, ^9^, ^10^ clinical PDLSC application is still far from the ultimate treatment goal for periodontitis: complete and functional regeneration of the periodontium in patients.^11^, ^12^ This limited efficacy may mainly be attributed to the low PDLSC survival rate and impaired cell potency/biological activity in the inflammatory and infectious microenvironment of lesion areas.^13^, ^14^, ^15^ Thus, further investigation of the fate and mechanisms of PDLSCs in the pathological microenvironment is crucial for their clinical application.

There are many different kinds cells and moleculars that can regulate periodontal healing/remodelling, of which macrophages are notable ones.^16^ It is well known that macrophages can promote wound healing and tissue regeneration by interaction with PDLSCs. The most widely known mechanism is that macrophages can polarize to distinct phenotypes and play corresponding roles by secreting corresponding cytokine, mediators and extracellular vesicles.^17^, ^18^ However, this is not the only way for macrophages to promote periodontal regeneration. Recently, the programmed cell death of macrophages such as apoptosis has been proved to beneficial for homeostatic cell turnover, cell proliferation and tissue regeneration.^19^ Thus, we speculate that pyroptosis, a kind of proinflammatory cell death, may play an essential role in tissue regeneration as well.

Pyroptosis features in the host response to pathogenic microorganism infection and cell stress.^20^ Canonical pathway of pyroptosis is characterized by the initiation of inflammasome‐associated caspases, maturation of IL‐1β and IL‐18, cleavage of gasdermin D (GSDMD) and pore formation in cell membrane. This process results in membrane rupture and leakage of cellular contents including cytokines and danger‐associated molecular patterns (DAMPs), which can initiate a strong inflammatory response.^21^, ^22^ Thus, pyroptosis serves as a line of defence against infections, including bacterial, fungal and viral invasions parallel to other innate immune responses.^23^, ^24^ However, dysregulated pyroptosis has been reported to participate in kinds of diseases, such as sepsis and cancer.^25^, ^26^ Accumulating evidences have also indicated that pyroptosis participates in periodontitis.^27^, ^28^, ^29^ During the pathophysiological process of periodontitis, pyroptosis occurs in kinds of cells, including macrophages, human gingival fibroblasts and PDLSCs.^27^, ^30^, ^31^ As a key mediator of the innate immune system, macrophages are the main sources of pyroptosis.^32^, ^33^ Moreover, overactivation of macrophages can expand the inflammatory response and tissue damage.^34^ Thus, further elucidation about the function of macrophage pyroptosis in periodontitis may help in its targeted treatment.

In recent years, many potential strategies targeting pyroptosis have been conducted to treat inflammatory diseases. Based on molecular mechanisms of pyroptosis, inhibitors and gene therapy have been attempted in preclinical experiments, for example, INF4E can directly affect NLRP3 activation, VX765 can regulate pyroptosis via caspase‐1 inhibition, knockout gene of the main executor GSDMD is also an effective way for pyroptosis control.^25^ However, the application of pyroptosis regulation in clinic still presents enormous challenges. Which cell type and pathway play the most important role in inflammation is still ambiguous. Due to the non‐specificity of pyroptotic inhibitors and the multiplicity of pyroptotic pathways, it is essential to enhance the specificity of inhibitors. What's more, the duration, dosage and administration method of inhibitor induction are unconfirmed, and that also makes the clinical utilization of inhibitors difficult. Moreover, excessive inhibition of cell pyroptosis can reduce the host's ability to resist pathogenic microorganisms, increasing the risk of inflammatory diseases. Thus, more strategies targeting pyroptosis are needed in further research. In previous studies, important molecular mechanisms have been found as hallmarks of inflammation, including transcriptional control, epigenetic modulations, metabolic regulations and etc.^35^ Particularly, the interplays between cellular metabolism and cell fate are being more appreciated in recent years. Metabolomics has been increasingly applied to disease diagnosis, including periodontal diseases. Several studies have reported the potential of metabolic profile to identify periodontal inflammation severity and therapeutic efficacy.^36^, ^37^ Even with all these efforts, currently, how the downstream metabolism pathways of pyroptosis regulate effector cell function transition remain incompletely understood.

In this study, we hypothesize that macrophage pyroptosis may damage the osteogenic differentiation potential of PDLSCs. We cultured PDLSCs with conditioned medium (CM) derived from pyroptotic macrophages and tested the osteogenic differentiation indexes. To reveal the underlying mechanism, we screened and identified the key metabolites associated with CM‐treated PDLSC under osteogenic differentiation incubation. This is the first report to uncover the effect of macrophage pyroptosis on the osteogenic differentiation of PDLSCs and the underlying metabolic mechanism. Our findings provide a new perspective for the regulation of osteogenic differentiation in the pathological microenvironment, which may be a novel and secure strategy for periodontal regeneration.

## 2 METHODS

### 2.1 Clinical specimens

Clinically healthy permanent teeth were obtained from adults (aged 18–25 years) for PDLSC culture. Gingival tissues were collected for immunofluorescence staining from healthy controls and patients suffering from periodontitis at stage I‐IV and grade C according to the latest classification.^38^, ^39^ The healthy group (8 donors, aged 18–45 years) consisted of donors who needed tooth extraction (impacted third molars or who required orthodontic therapy), or teeth requiring crown lengthening surgery. The patient group (periodontitis group, 9 donors, aged 18–45 years) consisted of patients requiring periodontal flap surgery. If there is more than one tooth meeting the inclusion criteria in a donor, one tooth is randomly selected for gingiva collection. The use of these extracted teeth and gingival tissues was approved by the ethics committee and institutional review board of the hospital, and all donors signed informed consent forms. The research protocol was consistent with the Declaration of Helsinki.

### 2.2 Immunofluorescence staining

Immunofluorescence labelling was conducted to study pyroptosis in macrophages. Gingival tissues were fixed in 4% paraformaldehyde (PFA; Invitrogen, CA, USA) for 48 h. After dealing with graded alcohols and dimethylbenzene, 3–5 μm paraffin embedded sections were prepared for staining. These sections were then deparaffinized, rehydrated and heated for antigen retrieval. After that, quenching reagent were added on sections, and then incubated with 10% goat serum for 30 min. Then incubated samples with anti‐CD68 (1:100; ab213363, Abcam, Cambridge, UK), anti‐caspase‐1 (1:25; 22915‐1‐AP, Proteintech Group, Chicago, USA) or anti‐GSDMD (1:50; 20770‐1‐AP, Proteintech) antibodies at 4°C overnight. After incubating with secondary antibody (1:50; 34806ES60, Yeasen, Shanghai, China) for 1 h, the slides were stained for 10 min with DAPI (1:1000; BD5010, Bioworld, Beijing, China) in the dark. A confocal laser microscope (Nikon A1 PLUS, Tokyo, Japan) was used to capture images.

### 2.3 Isolation and characterization of PDLSCs

Human PDLSCs were isolated from healthy teeth as reported previously.^18^, ^40^ Briefly, the periodontal ligament tissue was scraped from the tooth root, and then digested by collagenase type I (Merck, Darmstadt, Germany). Then, the digested tissue was resuspended in α‐MEM containing 10% fetal bovine serum and 1% penicillin/streptomycin. Then the digested tissue was incubated at 37°C, 5% CO_2_. After primary cells migrated from tissue pieces, they were seeded in 96‐well plates (density: 10–20 cells/mL) and expanded after colonies formed.

Human PDLSCs were identified by colony‐forming unit‐fibroblast (CFU‐F) assays, cell counting kit‐8 (CCK‐8) assays, flow cytometry, and multiple differentiation assays based on the ISCT definition of MSCs and studies reported previously.^18^, ^41^, ^42^, ^43^

#### 2.3.1 CFU‐F assay

PDLSCs were seeded and incubated in culture dishes (1 × 10^3^ cells per 10‐cm‐diameter dish) for 2 weeks. Then, the cells were stained with 0.1% crystal violet solution.

#### 2.3.2 CCK‐8 assay

For measurement of the proliferation of PDLSCs, cells were seeded in plates. CCK‐8 assays were carried out during a 7‐day culture period in line with the instructions of the CCK‐8 kit. After incubation with CCK‐8 reagent for 2 h, the optical density (OD) values were determined by a microplate reader at 450 nm (ELx800, BioTek, Vermont, USA).

#### 2.3.3 Flow cytometry analysis for immunophenotyping

Flow cytometry was used to determine the PDLSC immunophenotype. PDLSCs were collected into tubes (1 × 10^6^ cells per tube) and incubated with fluorescein isothiocyanate (FITC)‐/phycoerythrin (PE)‐conjugated antibodies against CD31, CD45, CD90, CD34, CD105 and CD146 (eBioscience, California, USA) for 1 h at 4°C. After phosphate buffered saline washes, the immunophenotypes of PDLSCs were assessed by flow cytometry (Beckman Counter, California, USA).

#### 2.3.4 Osteogenic differentiation assays

For osteogenic differentiation, PDLSCs were seeded in 6‐well plates or 12‐well plates (density: 1 × 10^5^ cells/mL) and cultured in osteogenic induction medium: complete α‐MEM with β‐glycerophosphate (10 mM), vitamin C (50 μg/mL) and dexamethasone (10 nM).

After 2 weeks of osteogenic induction, cell culture supernatants were collected. Alkaline phosphatase (ALP) assay kits (A059‐2, Nanjing Jiancheng Bioengineering, Nanjing, China) was used to determine ALP activity in supernatants. The absorbance (520 nm) was detected according to the kit instructions. Moreover, the cells in plates were fixed with 4% PFA, and then stained with an ALP stain kit (C3206, Beyotime, Shanghai, China).

After osteogenic induction for 3 weeks, the cells were fixed with 4% PFA and stained with 1% Alizarin Red S (ARS; ALIR‐10001, Cyagen, Guangzhou, China). Furthermore, a quantitative assay of the mineralized nodules was conducted. The stained areas were soaked in 2% cetylpyridinium chloride (H60001, Psaitong, Beijing, China) for 10 min. The absorbance of the solution was assessed at 562 nm.

#### 2.3.5 Adipogenic and chondrogenic differentiation assays

For adipogenic differentiation, PDLSCs were seeded in 12‐well plates (density: 1 × 10^5^ cells/mL) and cultured in adipoinductive medium (HUXMX‐90031, Cyagen). Then, the cells were stained with 0.3% Oil Red O (Merck) after adipogenic induction for 3 weeks.

For chondrogenic differentiation, PDLSCs were seeded into 15‐mL centrifuge tubes (3–4 × 10^5^ cells per tube) and cultured in chondrogenic induction medium according to the instructions of the kit (HUXMX‐90041, Cyagen). Then, Alcian blue (ALCB‐10001, Cyagen) staining and H&E staining were conducted after 4 weeks of culture.

### 2.4 Induction of macrophage pyroptosis and CM preparation

The THP‐1 cell line was gained from the American Type Culture Collection (ATCC TIB‐202) and cultured in RPMI 1640 medium. Before pyroptosis stimulation, THP‐1 cells were seeded into 6‐well plates (density: 7 × 10^5^ cells/mL) and induced into M0 macrophages by 320 nM phorbol 12‐myristate 13‐acetate (PMA; HY‐18739, MedChemExpress, New Jersey, USA) in serum‐free RPMI 1640 medium for 48 h.^18^ For pyroptosis stimulation, M0 macrophages were primed with 1 μg/mL lipopolysaccharide (LPS, Merck) for 3 h, followed by 10 μM nigericin (HY‐100381, MedChemExpresss) stimulation for 1 h in serum‐free RPMI 1640 medium. Additionally, in the pyroptotic inhibition group, cells were pretreated with 10 μM caspase‐1 inhibitor VX765 (HY‐13205, MedChemExpresss) for 0.5 h before priming with LPS and nigericin.

For M0 pyroptosis detection, the rate of pyroptotic cells were determined by flow cytometry. In addition, total cell lysates were collected for western blot analysis and caspase‐1 activity assay. For determination of the lactate dehydrogenase (LDH) activity and concentrations of IL‐1β released from M0, the culture supernatants were collected after LPS priming for 3 h and nigericin challenge for 4 h.

After pyroptosis stimulation, the culture supernatants were replaced with fresh serum‐free RPMI 1640 medium to remove stimuli (LPS, nigericin and VX765). The cells were subsequently incubated for another 3 h. Then, the supernatants were collected and centrifuged to remove cell residues as CM before being used to incubate PDLSCs.

#### 2.4.1 Flow cytometry analysis for pyroptosis

The pyroptotic stimulated M0 macrophages were collected and stained with an Annexin V‐FITC/PI kit (C1062, Beyotime). The two fluorescence signals were detected through the FITC channel (Annexin V‐FITC signal) and PE channel (PI signal) separately. Then, the rate of pyroptotic cells was analysed by the ratio of double‐positive cells to total cells.

#### 2.4.2 Caspase‐1 activity assay

Active caspase‐1 in cell lysates was measured by caspase‐1 activity assay kit (BC3810, Solarbio, Beijing, China). The absorbance was detected at 405 nm, and corrected with total protein.

#### 2.4.3 LDH assay

The LDH activity was detected using an LDH assay kit (A020‐2, Nanjing Jiancheng Bioengineering) according to the manufacturer's protocol. Then, the absorbance was recorded at 450 nm.

#### 2.4.4 Enzyme‐linked immunosorbent assay

Supernatants were measured for human IL‐1β based on the manufacturer's instructions of enzyme‐linked immunosorbent (ELISA) kits (SEKH‐0002, Solarbio). The absorbance was recorded at 450 nm.

### 2.5 Osteogenic induction of PDLSCs cocultured with pyroptotic macrophage‐derived CM

For determination of the effects of CM derived from pyroptotic M0 on the osteogenic differentiation of PDLSC, cells were plated in 6‐well or 12‐well plates (density: 1 × 10^5^ cells/mL) and cultured with CM‐LN, CM‐VX765 and CM‐M0 under osteogenic differentiation induction. One week later, quantitative real‐time polymerase chain reaction (qRT–PCR) and western blot were conducted to investigate the expression of osteogenic differentiation‐related genes and proteins in PDLSCs. ALP staining and ALP activity (2‐week induction), as well as ARS staining and quantitative assays of mineralized nodules (3‐week induction), were also conducted as mentioned above.

### 2.6 Western blot analysis

Total protein obtained from cell lysates was subjected to western blot analysis as previously reported.^43^, ^44^ The primary antibodies were used as follows: antibodies targeting caspase‐1 (1:1000; ab207802, Abcam), GSDMD (1:1000; ab210070, Abcam), IL‐1β (1:1000; ab216995, Abcam), COL1A1 (1:1000; ab138492, Abcam), ALPL (1:1000; ab65834, Abcam), RUNX2 (1:1000; 12556, Cell Signaling Technology, Boston, USA), OCN (1:1000; ab133612, Abcam), BMP2 (1:1000; 66383‐1‐Ig, Proteintech), SLC7A11 (1:1000; 43437, Signalway Antibody, Maryland, USA), GCLC (1:1000; 12601‐1‐AP, Proteintech), GCLM (1:1000; 14241‐1‐AP, Proteintech), and GSS (1:1000; 15712‐1‐AP, Proteintech). Horseradish peroxidase (HRP)‐conjugated secondary antibody (goat anti‐rabbit IgG; 1:10000; 31460, Thermo Fisher, Massachusetts, USA) was also used. For quantification of the protein expression levels, the grey values of bands were measured and then normalized using GAPDH (1:1000; 2118, Cell Signaling Technology).

### 2.7 qRT–PCR

Total RNA was extracted with RNAiso Plus (TaKaRa, Tokyo, Japan) and reverse‐transcribed to cDNA with Evo M‐MLV RT premix (Accurate Biotechnology, Hunan, China). Then qRT–PCR was conducted using commercial kits (Accurate Biotechnology) on a CFX96 Real‐time RT–PCR system (Bio‐Rad, California, USA). Table S1 is the primer sequences list. PCR results were normalized against an internal control (GAPDH).

### 2.8 Untargeted metabolomics based on ultra‐performance liquid chromatography–mass spectrometry (LC–MS)

PDLSCs were cultured with/without CM (derived from pyroptotic M0) under osteogenic differentiation induction for 1 week. Afterward, metabolite extraction was conducted as described with minor modifications. In brief, metabolites were collected from cells with 50% methanol buffer at −20°C. Then supernatants were collected for LC–MS analysis after centrifugation (4000 × *g*, 20 min). First, chromatographic separations were conducted by UltiMate 3000 HPLC (Thermo Fisher). In addition, reversed‐phase separation was conducted using an ACQUITY UPLC BEH C18 column (Waters, UK) at 35°C. The mobile phase rate was set at 0.4 mL/min, the mobile phase gradient setting was as below: 0 ~ 0.5 min, 5%; 0.5 ~ 7 min, 5% to 100%; 7 ~ 8 min, 100%; 8 min, 100% to 5%; and 8 ~ 10 min, 5%. Each sample was injected for 4 μL. The column effluent was then detected by Q Exactive (Thermo Fisher) in positive and negative modes. Moreover, precursor spectra (70–1050 m/z) was set at 70,000 resolutions, and the AGC target was 3 × 10^6^ ions. A quality control sample was also evaluated after every 10 samples.

Metabolomics data handling was performed using XCMS, CAMERA and metaX packages of R software. Metabolites were identified by m/z data and retention time, and annotated using the online KEGG database, HMDB and an in‐house fragment spectrum metabolites library. MetaboAnalyst 5.0 and the KEGG pathway database were used for metabolic pathway and function analysis.

### 2.9 Measurements of metabolite levels by kits

The levels of targeted metabolites were determined by commercial kits following the manufacturers' protocols. These kits included a glutamate measurement kit (A074‐1‐1, Nanjing Jiancheng Bioengineering), cysteine test kit (A126‐1‐1, Nanjing Jiancheng Bioengineering), oxidized glutathione (GSSG) assay kit (BC1185, Solarbio) and reduced glutathione (GSH) assay kit (BC1175, Solarbio). For all metabolite experiments, the quantity of metabolites was adjusted to the corresponding cell number or total protein concentration.

### 2.10 Statistical analysis

Experiments were replicated ≥3 times. Before conducting the data analysis, we identified and deleted extreme outliers (data exceeding mean value ±3 times standard deviation is defined outlier) in the raw data via statistical methods. Therefore, inconsistent sample sizes were observed between groups in some experiments. The continuous variables are showed as the mean ± standard deviation. Statistical analysis was determined by *t* test or one‐way ANOVA using GraphPad Prism 8. The two‐sided significance was set at *p < 0.05; **p < 0.01; ***p < 0.001.

For metabolomics analysis, principal component analysis (PCA) was used to compare the separation and aggregation between groups. The first and second principal component (PC1 and PC2) were obtained for PCA scores. However, partial least squares discriminate analysis (PLS‐DA) was used to distinguish the different variables between groups. VIP values were applied to filter differentially expressed metabolites (DEMs).

## 3 RESULTS

### 3.1 Macrophage pyroptosis is enriched in patients with periodontitis

**FIGURE 1:**
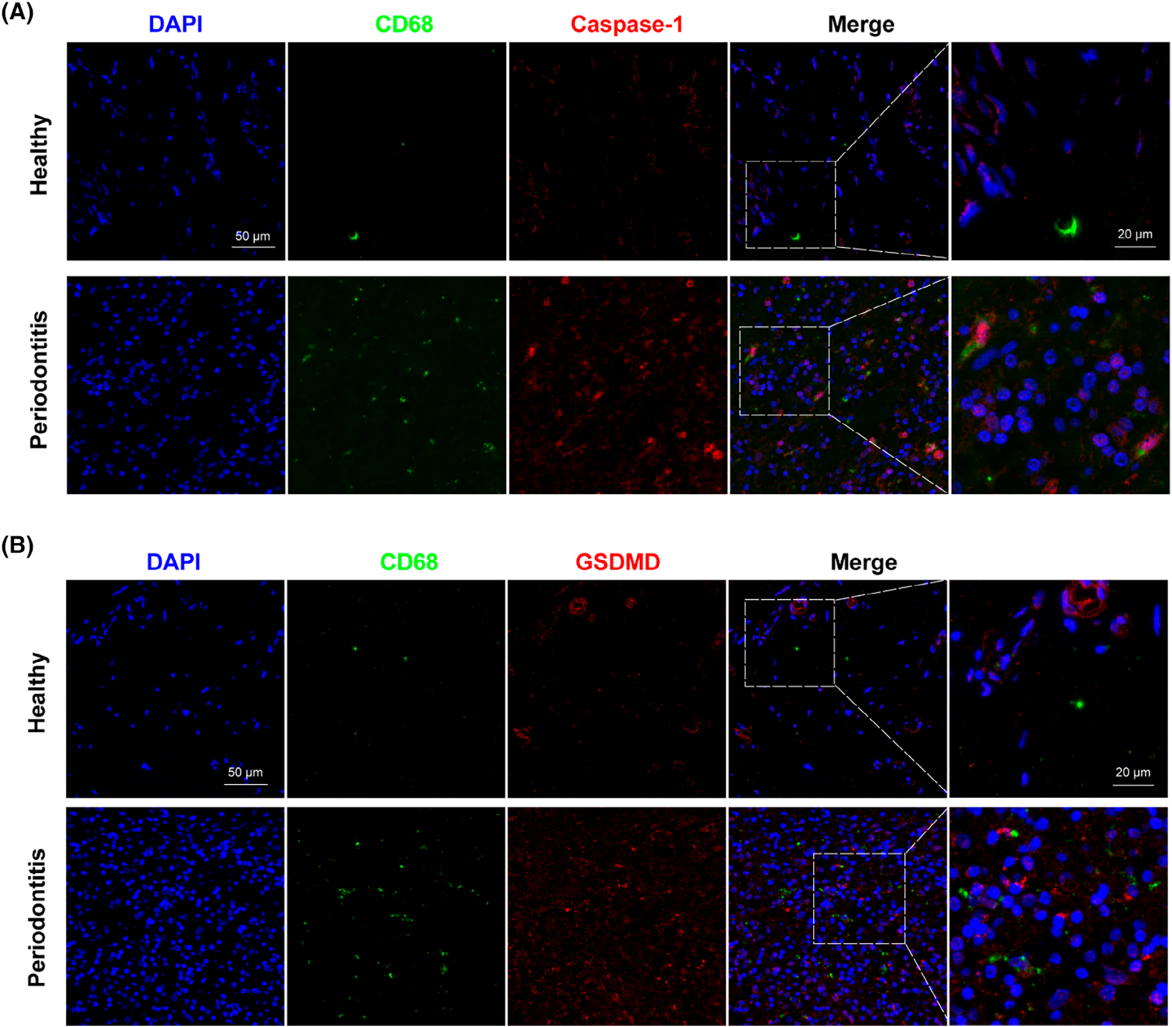
Macrophage pyroptosis is related with the inflammatory lesion of periodontitis. Human gingiva tissues were obtained from healthy individuals and patients with periodontitis. (A) Immunofluorescence staining of CD68 (green) and caspase‐1 (red). (B) Immunofluorescence staining of CD68 (green) and GSDMD (red). Nuclei were identified by DAPI (blue). Scale bar: 50 μm.

In this study, human gingival tissues were gotten from healthy humans and patients with severe periodontitis. Immunofluorescence was performed in human gingival tissues using a combination of CD68 and caspase‐1, CD68 and GSDMD to verify the presence of macrophage pyroptosis. As shown in Figure 1, the number of macrophages in the gingiva of patients with periodontitis was increased compared with that in healthy people. The co‐staining results of immunofluorescence showed that caspase‐1 (Figure 1A) and GSDMD (Figure 1B) were enriched in macrophages in patients with periodontitis. These results indicated that macrophage pyroptosis is related to inflammatory lesions in human periodontitis.

### 3.2 Macrophage pyroptosis compromises the osteogenic differentiation of PDLSCs

The results of the CCK‐8 assay confirmed the proliferative ability of the cells (Figure S1A). The spindle cells we isolated from human PDL tissue were plastic‐adherent and exhibited colony formation (Figure S1B). Flow cytometry analysis showed that these obtained cells positively expressed MSC markers but negatively expressed haematopoietic cell markers (CD31 and CD34) (Figure S1C). Furthermore, these cells had multiple differentiation potential. Mineralized nodules, lipid droplets and acidic polysaccharides were observed after osteogenic, adipogenic and chondrogenic induction, respectively (Figure S1D). These outcomes indicated that we successfully isolated human PDLSCs.

**FIGURE 2:**
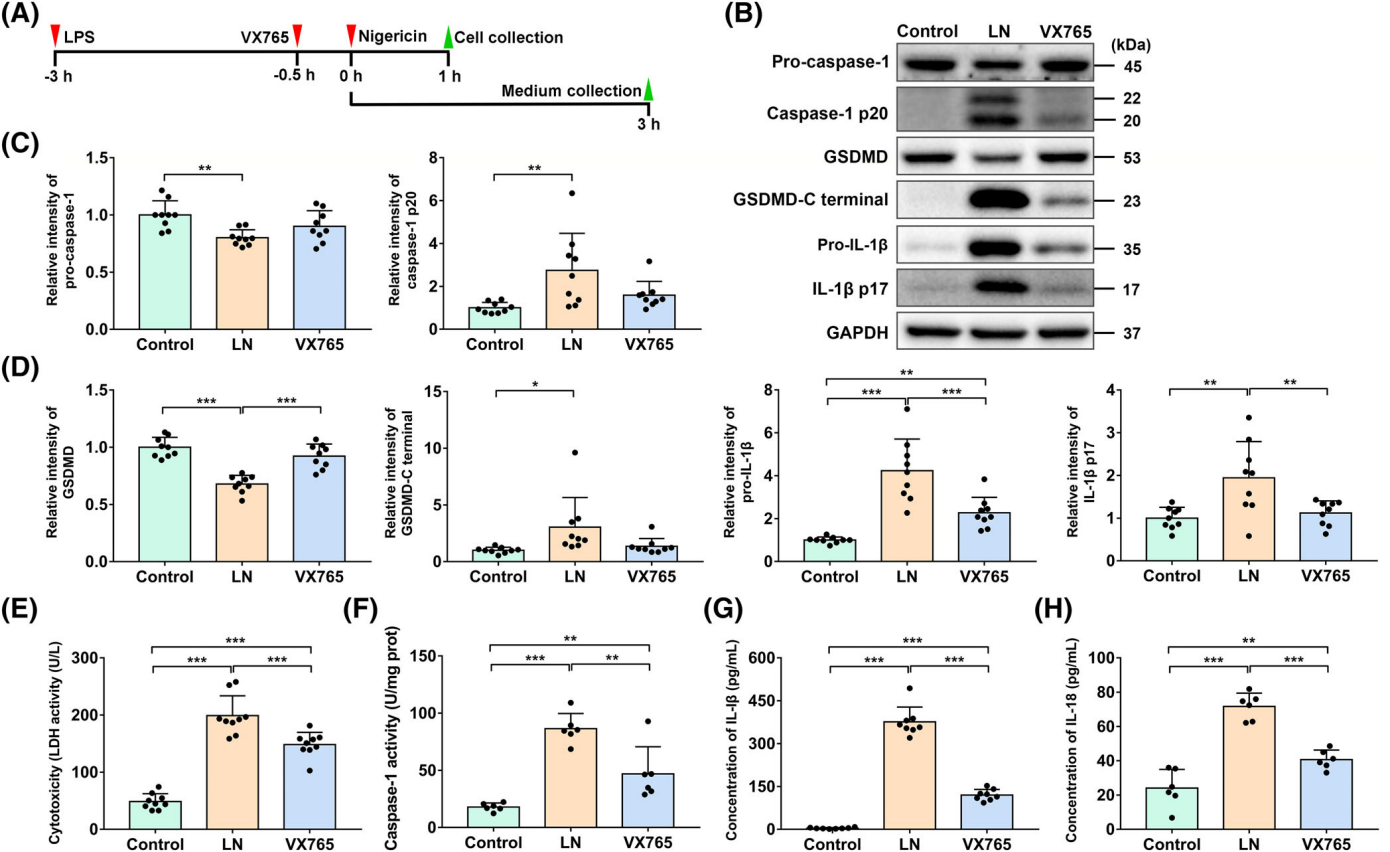
Induction and detection of macrophage pyroptosis. (A–H) Macrophages were set as control group, LPS and nigericin induced pyroptotic macrophages (LN group), and pyroptotic macrophages pretreated with VX765 (VX765 group). (A) Timeframe for macrophage pyroptosis induction. (B–D) The levels of GSDMD, GSDMD‐CT, pro‐caspase‐1, caspase‐1 p20, pro‐IL‐1β and IL‐1β p17 were detected by western blot. (E) LDH release was assayed by a commercial assay kit. (F) Caspase‐1 activity was detected by a commercial assay kit. (G, H) IL‐1β and IL‐18 secretion in cell culture supernatants were evaluated by ELISA. Data are shown as mean ± SD and analysed by one‐way ANOVA. Significant differences between two groups are shown by*p＜0.05； **p < 0.01; ***p < 0.001. If there is no marking on the graph, it means there is no statistical difference between two groups.

We first investigated whether pyroptosis occurred in M0 macrophages after pyroptosis stimulation (Figure 2A). Macrophages stimulated with LPS and nigericin, macrophages pretreated with VX765 (a caspase‐1 inhibitor) and stimulated by LPS and nigericin, and macrophages without any treatment were termed the LN group, VX765 group, and control group, respectively. The expression levels of pyroptosis‐related proteins in cells were analysed by western blot. The results showed that pyroptosis stimulation promoted caspase‐1 activation (pro‐caspase‐1 decreased and caspase‐1 p20 increased), GSDMD cleavage (GSDMD decreased and GSDMD‐CT increased) and IL‐1β maturation ( IL‐1β p17 increased) (Figure 2B–D). In addition, LDH release was increased in the culture supernatants of the LN group (Figure 2E). Caspase‐1 activity was also increased in cell lysates (Figure 2F). Moreover, IL‐1β and IL‐18 release were increased in the culture supernatants (Figure 2G,H). To further verify that pyroptosis stimulation was successful, we pretreated macrophages with VX765 prior to nigericin challenge (Figure 2A). As shown in Figure 2B,D,F, VX765 pretreatment alleviated IL‐1β maturation, GSDMD cleavage and caspase‐1 activation in pyroptotic macrophages. Moreover, VX765 inhibited the release of LDH, IL‐1β and IL‐18 into culture supernatants (Figure 2E,G,H). These results indicated the successful induction of macrophage pyroptosis.

**FIGURE 3:**
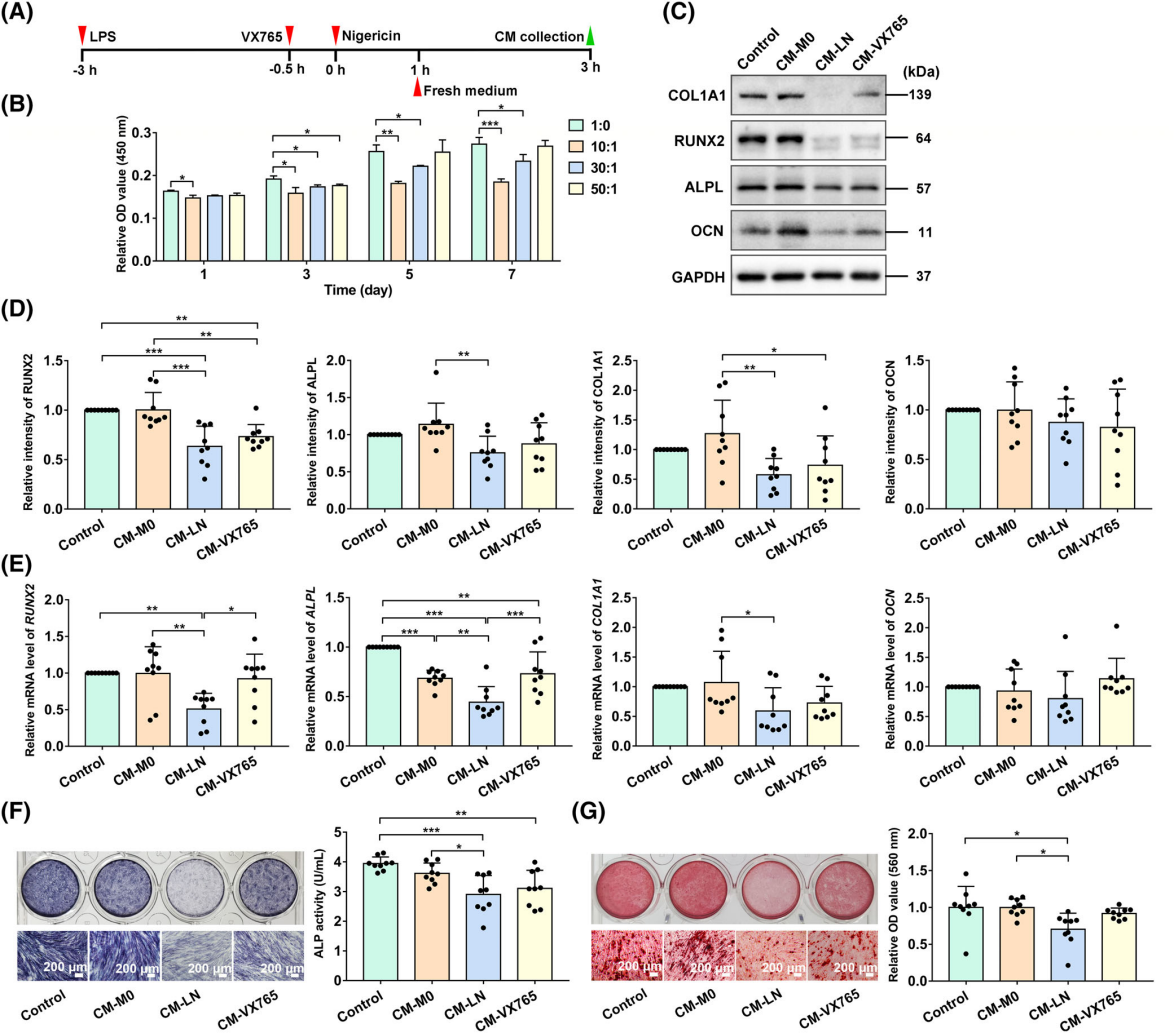
Conditioned medium (CM) derived from pyroptotic macrophages suppressed the osteogenic differentiation of PDLSCs. (A) Timeframe for CM preparation. (B) Screening a noncytotoxic concentration of CM by CCK‐8 assays. (C–E) PDLSCs were cultured in the mixture (volume ratio at 50:1) of osteogenic induction medium and CMs derived from supernatants of pyroptotic macrophages, VX765 pretreated pyroptotic macrophages and macrophages without any treatment, and these PDLSCs were termed as CM‐LN group, CM‐VX765 group and CM‐M0 group, respectively. Serum‐free RPMI 1640 medium (equal volume with other CMs) was added as control. (C, D) Western blot for relative osteogenic differentiation‐related protein levels of COL1A1, ALPL, RUNX2 and OCN in cell lysates. (E) Relative gene expression of *COL1A1*, *ALPL*, *RUNX2* and *OCN* determined by qRT‐PCR. (F) ALP staining (scale bar: 200 μm) and cellular ALP activity assays by commercial assay kits. (G) Alizarin Red staining (scale bar: 200 μm) and quantitative assays. Data are shown as mean ± SD and analysed by one‐way ANOVA. Significant differences between two groups are represented by *p < 0.05; **p < 0.01; ***p < 0.001. If there is no marking, it means there is no statistical difference.

To investigate the effect of pyroptotic M0 macrophages on the osteogenic differentiation of PDLSCs, we used a CM‐based coculture system (Figure 3A). We first screened a noncytotoxic concentration of CM by CCK‐8 assays. PDLSCs were cultured in a mixture of complete α‐MEM and CM (derived from pyroptotic macrophages) at different volume ratios (1:0, 10:1, 30:1, 50:1). Considering the cell viability determined at 1, 3, 5 and 7 days, the 50:1 concentration was selected for the following investigation (Figure 3B). PDLSCs were then cultured in a mixture (volume ratio at 50:1) of osteogenic induction medium and CM derived from supernatants of the LN group, VX765 group and M0 group, and these PDLSCs were termed the CM‐LN group, CM‐VX765 group and CM‐M0 group, respectively. Moreover, serum‐free RPMI 1640 medium (equal volume with other CMs) was added to PDLSCs under osteogenic induction as a control. After osteogenic incubation, the CM‐LN group expressed markedly lower levels of osteogenic differentiation‐related proteins (COL1A1, ALPL and RUNX2) and genes (*COL1A1*, *ALPL* and *RUNX2*) than the CM‐M0 group (Figure 3C–E). In addition, positively stained PDLSCs (ALP staining) and cellular ALP activity were decreased in the CM‐LN group (Figure 3F). ARS staining of mineralized nodules and quantitative analysis also demonstrated reduced osteogenic differentiation potential of the CM‐LN group compared with the CM‐M0 group and control group (Figure 3G). Besides, osteogenic differentiation‐related gene levels (*ALPL* and *RUNX2*) in the CM‐VX765 group were increased compared with those in the CM‐LN group (Figure 3C–G). These results indicate that pyroptotic M0 macrophages may damage the osteogenic differentiation of PDLSCs, while inhibiting M0 pyroptosis may ameliorate osteogenic inhibition.

### 3.3 Screening for the key metabolite involved in CM‐inhibited osteogenic differentiation

**FIGURE 4:**
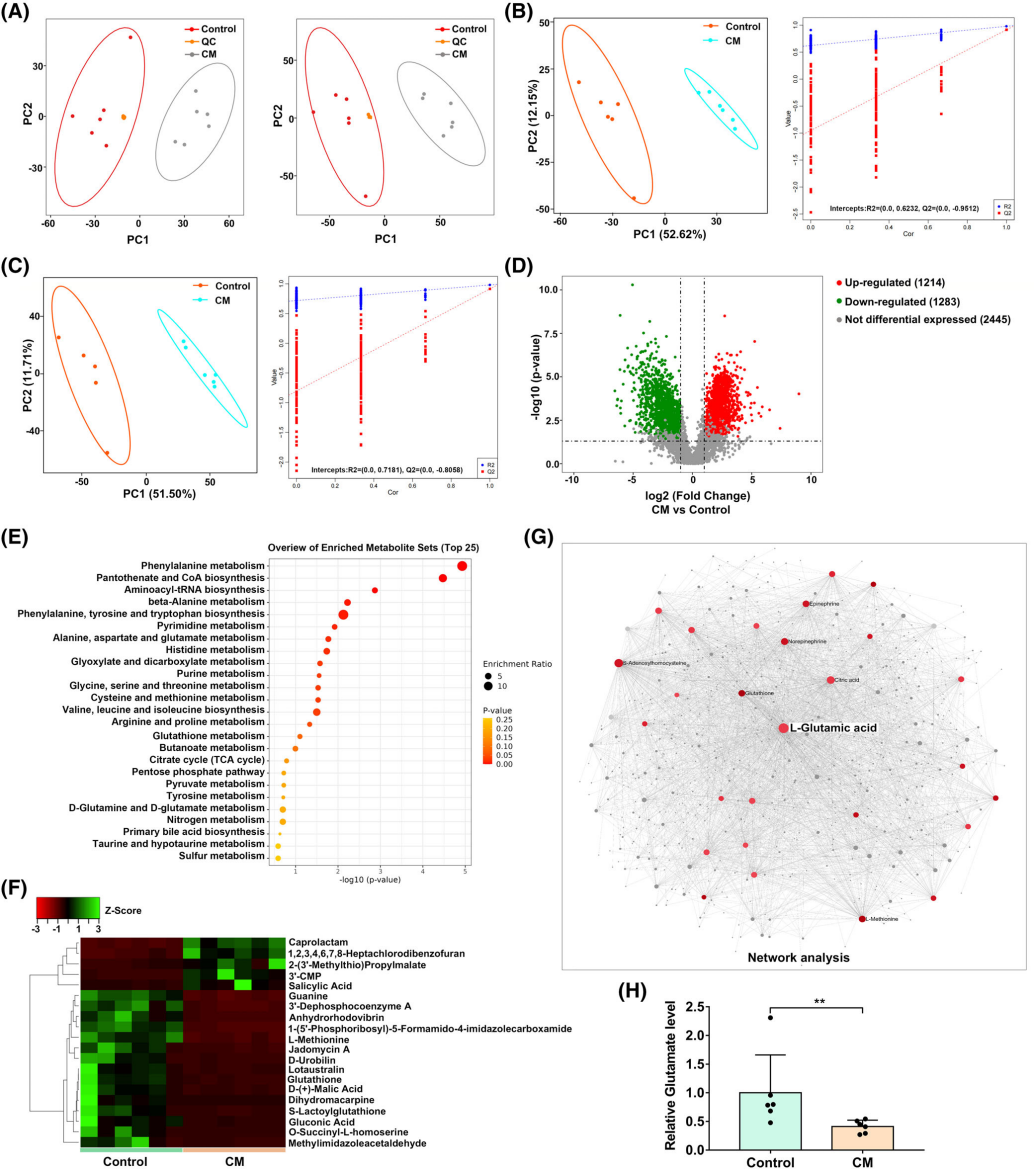
Screening for the key metabolite involved in conditioned medium (CM)‐inhibited osteogenic differentiation. (A) Principal components analysis (PCA) in negative mode (left) and positive mode (right). (B) Partial least squares discriminate analysis (PLS‐DA) model and permutation testing in negative mode. (C) PLS‐DA model and permutation testing in positive mode. (D) The volcano plots showing DEMs between control and CM‐treated PDLSCs. (E) KEGG enrichment analysis. (F) The heatmap of the top 20 regulated metabolites in CM‐treated PDLSCs compared with the control group. The colour from green to red indicated high to low metabolite levels. (G) Network analysis of DEMs. (H) Glutamate levels between the CM‐treated PDLSCs and the control group by commercial kits. Significant difference between the two groups is represented by **p < 0.01.

Metabolomic profiling based on LC–MS/MS was conducted to identify DEMs between PDLSCs cocultured with CM and those without CM in osteogenic induction medium. A total of 4942 metabolites were identified. The PCA (Figure 4A) and PLS‐DA (Figure 4B,C) models demonstrated a significant separation trend between the two group. Based on the requirements of |log_2_FC| > 1, *p* < 0.05 and VIP > 1, a total of 2497 metabolites (1214 increased, 1283 decreased) were selected as DEMs between the two groups, as shown in the volcano plots (Figure 4D). Among these DEMs, only 111 metabolites could be annotated. To further identify relevant metabolic pathways, we performed KEGG enrichment analysis. Metabolic pathways involving phenylalanine metabolism, pantothenate and CoA biosynthesis, aminoacyl‐tRNA biosynthesis, and other pathways were the top targets (Figure 4E). A heatmap was drawn to demonstrate the changes in the top 20 metabolites in CM‐treated PDLSCs (Figure 4F). The top 10 regulated metabolites were reported in Table S2. Then, a network analysis of all annotated DEMs was conducted using MetaboAnalyst 5.0. The top 10 central nodes of the DEMs are listed in Table S3. And the results showed that glutamate was in the centre of the DEM network (Figure 4G). Glutamate levels between the CM‐treated PDLSCs and the control group were further verified based on assay kits (Figure 4H). Thus, we speculated that glutamate may be a key metabolite for CM‐inhibited osteogenesis of PDLSCs.

### 3.4 Exogenous glutamate aggravates the CM‐inhibited osteogenic differentiation of PDLSCs

**FIGURE 5:**
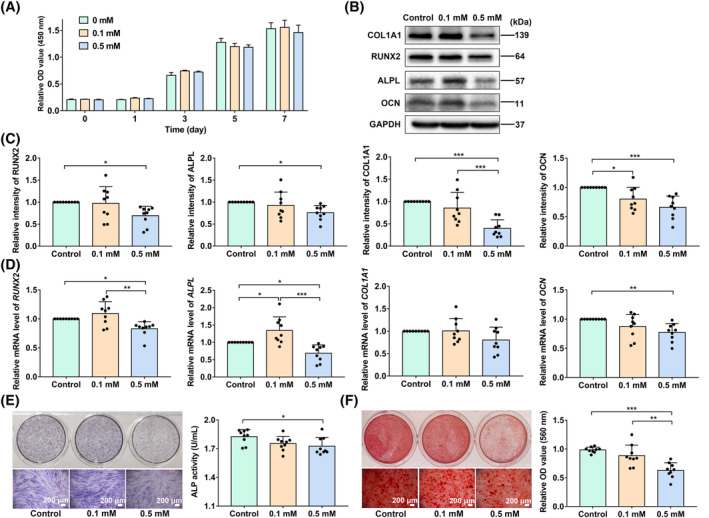
Exogenous glutamate aggravated the conditioned medium (CM)‐inhibited osteogenic differentiation of PDLSCs. (A) The cytotoxicity of selected concentrations of glutamate by CCK‐8 assays. (B–E) CM‐LN treated PDLSCs were divided into three groups, two of which were supplemented with 0.1 and 0.5 mM glutamate separately, with the remaining group served as control group. (B, C) Western blot for osteogenic differentiation‐related protein levels of COL1A1, ALPL, RUNX2 and OCN. (D) Relative expressions of osteogenic differentiation‐related genes *COL1A1*, *ALPL*, *RUNX2* and *OCN* analysed by qRT‐PCR. (E) ALP staining (scale bar: 200 μm) and cellular ALP activity assays. (F) Alizarin Red staining (scale bar: 200 μm) and quantitative assays. Data are shown as mean ± SD and analysed by one‐way ANOVA. Significant differences between two groups are represented by *p < 0.05; **p < 0.01; ***p < 0.001. If there is no marking, it means there is no statistical difference.

The concentrations of glutamate were set based on previous reports.^45^, ^46^ Seidlitz EP and colleagues treated osteoblasts with 0.1 and 0.3 mM glutamate, and osteoclasts with 0.1 and 0.5 mM glutamate.^45^ Takarada‐Iemata M and colleagues treated osteoblasts with 0.01, 0.1, 0.5 and 1 mM glutamate.^46^ Therefore, we selected 0.1 mM and 0.5 mM glutamate to study the effect of glutamate on PDLSCs. The cytotoxicity of glutamate was studied by CCK‐8 assays. Based on the cell viability, the two concentrations of glutamate were confirmed to have no cytotoxicity and could be used for the following investigations (Figure 5A). In the CM treated PDLSCs, the intracellular glutamate level was increased by the exogenous addition of 0.5 mM glutamate (Figure S2). To observe whether glutamate can rescue CM‐inhibited PDLSC osteogenesis, we divided CM‐treated PDLSCs into three groups, two of which were separately administered 0.1 and 0.5 mM glutamate in the culture medium, with the remaining group serving as the control group. Then, the three groups were conducted to osteogenic induction. The results showed that the expression levels of proteins (COL1A1, ALPL, RUNX2 and OCN) and genes (*ALPL*, *RUNX2* and *OCN*) in PDLSCs were markedly decreased after glutamate (0.5 mM) treatment compared with those of the control group (Figure 5B–D). Moreover, similar results were also observed in ALP staining and ALP activity assays (Figure 5E), as well as ARS staining and quantitative analysis (Figure 5F). The results above showed that the addition of exogenous glutamate aggravates their compromised osteogenic differentiation.

While adding glutamate to the CM treated PDLSCs, we also verified the effects of glutamate on PDLSCs cultured in normal medium (Figure S3). The results showed that glutamate had no statistically significant effect on the osteogenic differentiation of PDLSCs.

### 3.5 CM derived from pyroptotic macrophages increases the consumption of glutamate in PDLSCs

**FIGURE 6:**
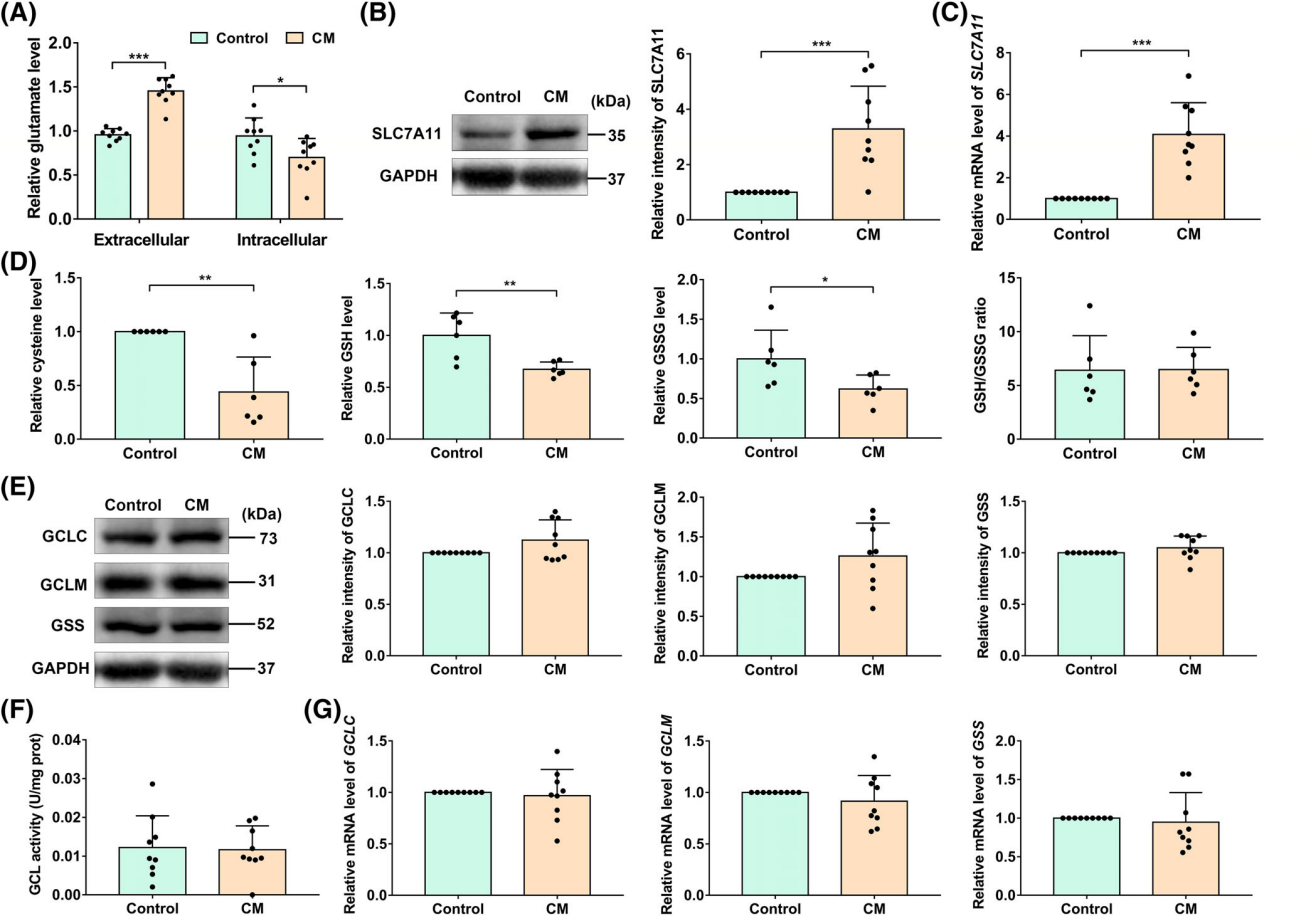
Conditioned medium (CM) promoted the PDLSC extracellular transport of glutamate. (A) The concentrations of intracellular and extracellular glutamate. (B, C) Gene and protein levels of SLC7A11. (D) The levels of intracellular cysteine, GSH, GSSG and GSH/GSSG ratio with/without CM‐treatment. (E) Protein levels of GCLC, GCLM and GSS. (F) Activity of GCL. (G) Gene levels of *GCLC*, *GCLM* and *GSS*. Data are shown as mean ± SD and analysed by *t* test. Significant differences between two groups are shown by *p < 0.05; **p < 0.01; ***p < 0.001. If there is no marking, it means there is no statistical difference.

With the help of commercial kits, we confirmed that the concentration of extracellular glutamate was increased, while the intracellular glutamate was decreased significantly in PDLSCs after CM treatment (Figure 6A). These interesting results indicate that CM increased the extracellular transport of glutamate in PDLSCs. System x_c_^−^ is a concentration dependent antiporter that can transport glutamate out of cells and cystine into cells. Cysteine is converted from cystine and is a rate‐limiting substrate for GSH synthesis. Thus, we detected the gene and protein levels of SLC7A11, the main functional unit of system x_c_^−^, and found that they were both increased (Figure 6B,C). What's more, the levels of intracellular cysteine, GSH and GSSG were all significantly decreased (Figure 6D). Therefore, we speculate that glutamate, cystine and its downstream product GSH may play a significant role in CM‐inhibited osteogenesis. We then detected the functions of the GSH synthetase glutamate cysteine ligase (GCL) and glutathione synthetase (GSS). The protein levels of GCLC, GCLM and GSS, the activity of GCL, and the gene levels (*GCLC*, *GCLM* and *GSS*) did not change with CM treatment (Figure 6E–G). Thus, we conclude that the compromised PDLSCs may be caused by their increased consumption of cystine and its downstream metabolites, which results in the abnormal transport of glutamate.

### 3.6 Increased endogenous glutamate induced by cystine rescues CM‐inhibited osteogenesis of PDLSCs

**FIGURE 7:**
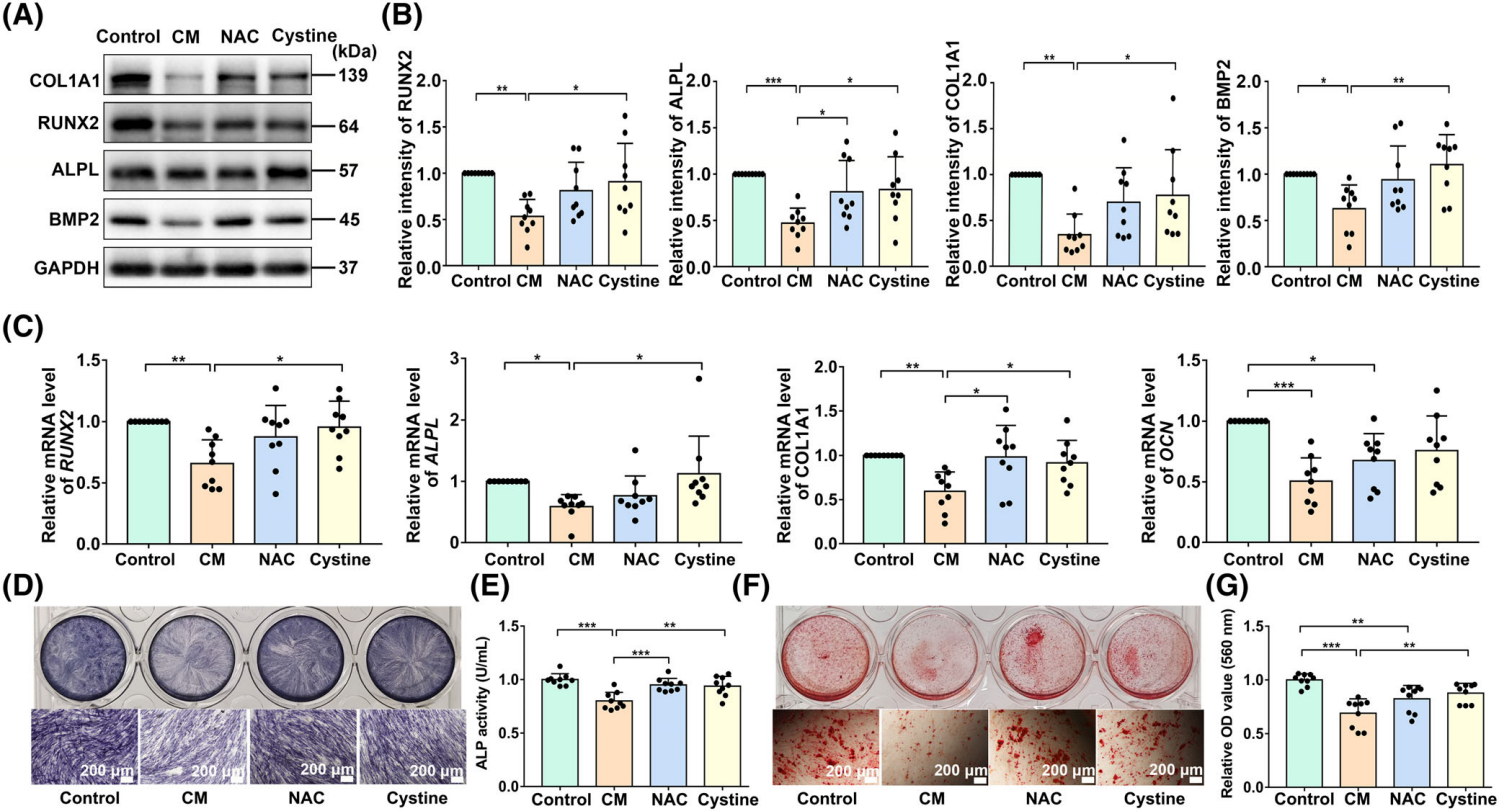
Cystine rescued conditioned medium (CM)‐inhibited osteogenesis of PDLSCs. (A–G) CM‐treated PDLSCs were divided into three groups, two of which were supplemented with 0.5 mM cystine and 0.5 mM NAC separately (cystine group and NAC group), the remaining group did not supplement with any reagent (CM group). PDLSCs not treated with CM were set as the control group. (A, B) Western blot for relative protein levels of COL1A1, ALPL, RUNX2 and BMP2. (C) Relative expressions of osteogenic differentiation‐related genes *COL1A1*, *ALPL*, *RUNX2* and *OCN* analysed by qRT‐PCR. (D) ALP staining (scale bar: 200 μm). (E) Cellular ALP activity assays. (F) ARS staining (scale bar: 200 μm). (G) Quantitative assays of ARS staining. Data are shown as mean ± SD and analysed by one‐way ANOVA. Significant differences between two groups are shown by *p < 0.05; **p < 0.01; ***p < 0.001. If there is no marking, it means there is no statistical difference.

To observe whether cystine can rescue CM‐inhibited PDLSC osteogenesis, we divided CM‐treated PDLSCs into three groups, two of which were separately administered 0.5 mM cystine and 0.5 mM N‐Acetylcysteine (NAC) (cystine group and NAC group), with the remaining group did not supplement with any reagent (CM group). PDLSCs not treated with CM were set as the control group. Then, the four groups were subjected to osteogenic induction. With cystine supplement, the expression levels of osteogenic differentiation‐related proteins (COL1A1, ALPL, RUNX2 and BMP2) and genes (*COL1A1*, *ALPL* and *RUNX2*) were markedly increased compared with the CM‐treated PDLSCs (Figure 7A–C). Similar results were also observed in the ALP staining (Figure 7D) and ALP activity assays (Figure 7E), as well as ARS staining and quantitative analysis (Figure 7F,G). The results also showed that NAC supplement increased osteogenic differentiation‐related protein levels of ALPL, gene level of *COL1A1*, and ALP activity (Figure 7A–E).

## 4 DISCUSSION

In recent years, many studies have reported that pyroptosis plays an important role in many tissue‐destructive diseases, including periodontitis.^25^, ^27^ Attachment and bone loss in periodontitis are the results of the host immune response to bacterial infection and its metabolic byproducts.^1^ As a type of proinflammatory cell death, pyroptosis can initiate a strong inflammatory response via the rapid release of large amounts of cellular contents.^47^ These cellular contents, such as IL‐1β and HMGB1, have been reported to regulate migration, proliferation and differentiation of stem cells.^48^, ^49^, ^50^ We found that CM derived from pyroptotic macrophages damaged the osteogenic differentiation of PDLSCs in this study.

As is well known, macrophage plays a vital role in the development of periodontitis. Macrophages can rapidly proliferate, migrate, and accumulate when inflammation occurs.^34^, ^51^ Therefore, this study (Figure 1) showed that the number of macrophages in the periodontitis group was much greater than that in the healthy group. Furthermore, the absolute number of pyroptotic macrophages in the periodontitis group was much greater than that in the healthy group, reflecting the significance of pyroptotic macrophages in periodontitis.

Similar to other studies, our study used the human THP‐1 monocyte cell line to obtain macrophages owing to its convenient expansion and reproducible results ex vitro.^52^, ^53^ Studies have shown that stem cells can regulate macrophage pyroptosis. Instead of using the transwell system to explore the influence of macrophages on stem cells, we collected the culture supernatant of macrophages and cocultured them with PDLSCs. In addition, to eliminate the effect of stimuli, including LPS and nigericin, on PDLSCs, we refreshed the culture medium before CM collection. There are many methods for inducing cell pyroptosis, such as cytosolic LPS, bacterial type III SA, flagellin and ATP.^54^ LPS and nigericin were used to induce canonical pyroptosis based on previous studies.^27^, ^55^, ^56^ Although the results of GSDMD cleavage, caspase‐1 initiation and cell lysis proved the occurrence of macrophage pyroptosis, the caspase‐1‐specific inhibitor VX765 did not completely inhibit the initiation of caspase‐1 and the release of LDH, IL‐1β and IL‐18 (Figure 2). In fact, LPS participates not only in pyroptosis but also in necroptosis. The two kinds of programmed cell death are lytic and inflammatory cell death, which can release numerous DAMPs and trigger inflammatory responses.^54^, ^57^ Moreover, there is crosstalk between pyroptosis and necroptosis; for example, MLKL (a central player in necroptosis) has been reported to be related with NLRP3/caspase‐1 activation.^57^ Therefore, it is difficult to completely distinguish the two types of cell death, which is also the dilemma of this study. Another problem is the polarization of macrophages. Studies have reported the presence of macrophage polarization when inducing pyroptosis.^58^, ^59^ Macrophages can polarize into kinds of phenotypes, such as M1 and M2 macrophages, under microenvironmental stimuli.^60^ These polarized macrophages can secrete cytokines such as IL‐1β, IL‐10 and TNF‐α and then regulate the inflammation and behaviour of stem cells.^17^, ^18^, ^34^ Consequently, we speculate that when we induce macrophage pyroptosis, other biological processes, such as necroptosis and macrophage polarization, also occur. In fact, more cell death types, including necroptosis, apoptosis, NETosis, and pyroptosis, are usually concurrent in pathological status. Identifying which type of cell death is predominant in diseases may help us to develop more targeted and precise treatments.

When pyroptosis occurs, the cell contents such as LDH, HMGB1, IL‐1 family (the highly inflammatory cytokines), and other cellular danger signals (DAMPs) are released.^21^, ^22^ Previous studies found that IL‐1β acts as a major cytokine released in pyroptotic PDLSCs, and regulates osteogenic and osteoclastogenic differentiation.^27^ Further evidence is needed to determine which kind of DAMP has the greatest impact on downstream effector cells.

Pyroptosis is a double‐edged sword for humans. Thus, research on the functions of pyroptosis has mainly focused on two aspects. On the one hand, by inducing pyroptotic tumour cell death, pyroptosis can be used to destroy cancers. By recruiting many immune cells, rapid and intense activation of pyroptosis can also provoke antitumor immunity and restrain the further growth of tumours.^61^, ^62^ On the other hand, excessive inflammatory responses caused by pyroptosis may result in tissue destruction, such as periodontitis, liver diseases, and nervous system diseases. Therefore, researchers are trying to inhibit key players (such as GSDMD, NLRP3 or caspase‐1) in the pyroptosis pathway to restrict inflammation and reduce tissue destruction. For example, VX‐765 is a caspase‐1 inhibitor that has been proven effective and well tolerated in patients with partial epilepsy. Similarly, in this study, we pretreated pyroptotic macrophages with VX765 and verified its inhibitory effect on macrophage pyroptosis and protective effect on PDLSC osteogenic differentiation (Figure 3). The therapeutic strategy targeting key molecules of pyroptosis may be a novel way to address inflammatory diseases associated with pyroptosis. However, because of non‐tissue specificity, the clinical treatment of pyroptosis will cause uncontrollable side effects. For example, normal tissue cells may be killed simultaneously when activating pyroptosis to clear cancers; the innate immune responses of the host may also be limited when inhibiting pyroptosis to treat immune or inflammatory diseases. There is still a long way to go for the clinical treatment and application of pyroptosis.

PDLSCs are promising ‘seeds’ for periodontal tissue engineering and regeneration, and how to maintain their biological activity in the inflammatory microenvironment is crucial for periodontitis treatment. We found that pyroptotic macrophages inhibited the osteogenic differentiation of PDLSCs (Figure 3) in this study. Due to the uncontrollability of targeted pyroptosis treatment, targeted therapy for downstream PDLSCs may be a promising strategy. Our study indicated that nutrient starvation (mainly cystine) is one of the reasons for osteogenic differentiation damage in PDLSCs. Cysteine is an important substrate for the synthesis of glutathione. However, further investigations are needed to clarify the mechanism of cystine starvation. One possible reason is the redox imbalance and the increased consumption of glutathione under the influence of CM. Another reason may be the reduction in cystine generation.

**FIGURE 8:**
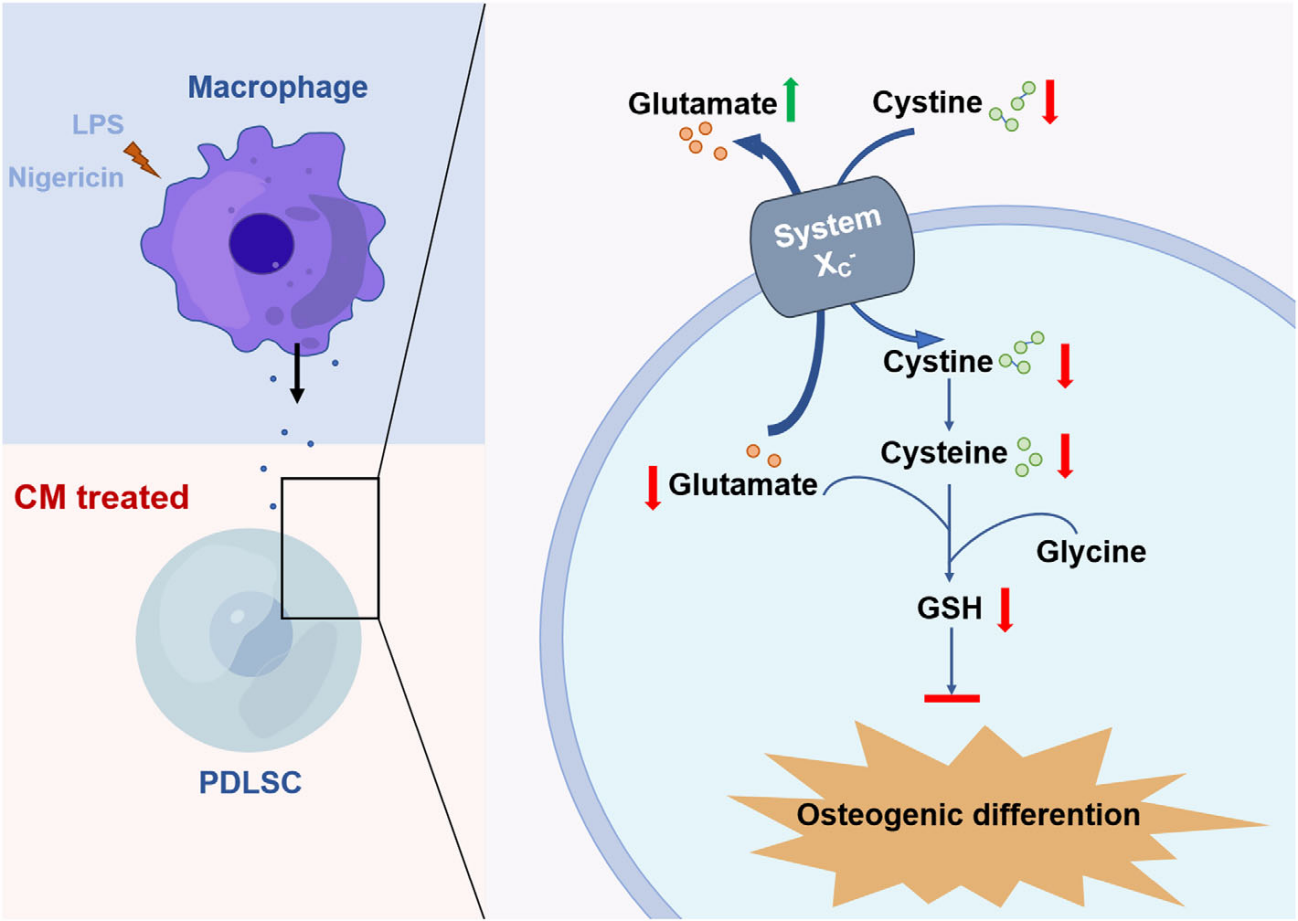
The diagram shows that glutamate plays a central role in regulating osteogenic differentiation of periodontal ligament stem cells compromised by pyroptotic macrophages.

To our knowledge, it is the first study to reveal the effect of macrophage pyroptosis on PDLSC osteogenic differentiation via glutamate metabolism disruption (Figure 8). Our findings will help pave the way for periodontitis and periodontal regeneration based on stem cell‐based therapy. In contrast to previous strategies to change the fate of stem cells, for example, using drugs, changing the environment of cells, gene therapy and other methods, our research found that supplementation with nutrients may also improve the performance of stem cells in the inflammatory microenvironment.

## 5 CONCLUSIONS

Our findings suggest that macrophage pyroptosis may play an important role in the progression of inflammatory lesions in periodontitis by inhibiting the osteogenic differentiation of PDLSCs. Either pharmacological inhibition of macrophage pyroptosis or nutritional supplements to PDLSCs, such as cystine, can rescue the compromised cell osteogenic potency caused by pyroptotic macrophages.

## AUTHOR CONTRIBUTIONS

All authors approved the final version of manuscript. Li‐Juan Sun and Hong‐Lei Qu conceptualized the study, performed the experiments and prepared the manuscript. Xiao‐Tao He, Bei‐Min Tian, Rui‐Xin Wu, Yuan Yin and Jie‐Kang Zou helped with performing experiments. Bei‐Min Tian and Hai‐Hua Sun provided financial support. Xuan Li and Fa‐Ming Chen supervised the study, provided financial support and revised the manuscript.

## FUNDING INFORMATION

We acknowledge the fundings supported by the Major Research Program of the National Natural Science Foundation of China (Subproject No. 82130026), the National Natural Science Foundation of China (Subproject Nos. 82170926, 82301079, 82170958 and 82370957), the Young Elite Scientist Sponsorship Program by CAST (Subproject No. 2022QNRC001) and the China Postdoctoral Science Foundation (Subproject No. 2022TQ0134).

## CONFLICT OF INTEREST STATEMENT

The authors declare no competing interests.

## Supporting information


**Data S1:** Supporting Information.

## Data Availability

The datasets for metabolomics analysis during the current study are available in MetaboLights (https://www.ebi.ac.uk/metabolights/) [ID: MTBLS9204].

## References

[1] Kinane DF , Stathopoulou PG , Papapanou PN . Periodontal diseases. Nat Rev Dis Primers. 2017;3:17038. doi:10.1038/nrdp.2017.38 28805207

[2] Hussain SB , Botelho J , Machado V , et al. Is there a bidirectional association between rheumatoid arthritis and periodontitis? A systematic review and meta‐analysis. Semin Arthritis Rheum. 2020;50(3):414‐422. doi:10.1016/j.semarthrit.2020.01.009 32113837

[3] Muñoz Aguilera E , Suvan J , Buti J , et al. Periodontitis is associated with hypertension: a systematic review and meta‐analysis. Cardiovasc Res. 2020;116(1):28‐39. doi:10.1093/cvr/cvz201 31549149

[4] Teeuw WJ , Gerdes VE , Loos BG . Effect of periodontal treatment on glycemic control of diabetic patients: a systematic review and meta‐analysis. Diabetes Care. 2010;33(2):421‐427. doi:10.2337/dc09-1378 20103557 PMC2809296

[5] Chen FM , Wu LA , Zhang M , Zhang R , Sun HH . Homing of endogenous stem/progenitor cells for in situ tissue regeneration: promises, strategies, and translational perspectives. Biomaterials. 2011;32(12):3189‐3209. doi:10.1016/j.biomaterials.2010.12.032 21300401

[6] Fu J , Wang Y , Jiang Y , Du J , Xu J , Liu Y . Systemic therapy of MSCs in bone regeneration: a systematic review and meta‐analysis. Stem Cell Res Ther. 2021;12(1):377. doi:10.1186/s13287-021-02456-w 34215342 PMC8254211

[7] Han Y , Li X , Zhang Y , Han Y , Chang F , Ding J . Mesenchymal stem cells for regenerative medicine. Cells. 2019;8(8):886. doi:10.3390/cells8080886 31412678 PMC6721852

[8] Vaquette C , Saifzadeh S , Farag A , Hutmacher DW , Ivanovski S . Periodontal tissue engineering with a multiphasic construct and cell sheets. J Dent Res. 2019;98(6):673‐681. doi:10.1177/0022034519837967 30971166

[9] Menicanin D , Mrozik KM , Wada N , et al. Periodontal‐ligament‐derived stem cells exhibit the capacity for long‐term survival, self‐renewal, and regeneration of multiple tissue types in vivo. Stem Cells Dev. 2014;23(9):1001‐1011. doi:10.1089/scd.2013.0490 24351050 PMC3997143

[10] Tassi SA , Sergio NZ , Misawa MYO , Villar CC . Efficacy of stem cells on periodontal regeneration: systematic review of pre‐clinical studies. J Periodontal Res. 2017;52(5):793‐812. doi:10.1111/jre.12455 28394043

[11] Chen FM , Gao LN , Tian BM , et al. Treatment of periodontal intrabony defects using autologous periodontal ligament stem cells: a randomized clinical trial. Stem Cell Res Ther. 2016;7:33. doi:10.1186/s13287-016-0288-1 26895633 PMC4761216

[12] Hoang DM , Pham PT , Bach TQ , et al. Stem cell‐based therapy for human diseases. Signal Transduct Target Ther. 2022;7(1):272. doi:10.1038/s41392-022-01134-4 35933430 PMC9357075

[13] Bogdanowicz DR , Lu HH . Designing the stem cell microenvironment for guided connective tissue regeneration. Ann N Y Acad Sci. 2017;1410(1):3‐25. doi:10.1111/nyas.13553 29265419

[14] Merimi M , El‐Majzoub R , Lagneaux L , et al. The therapeutic potential of mesenchymal stromal cells for regenerative medicine: current knowledge and future understandings. Front Cell Dev Biol. 2021;9:661532. doi:10.3389/fcell.2021.661532 34490235 PMC8416483

[15] Silva LHA , Antunes MA , Dos Santos CC , Weiss DJ , Cruz FF , Rocco PRM . Strategies to improve the therapeutic effects of mesenchymal stromal cells in respiratory diseases. Stem Cell Res Ther. 2018;9(1):45. doi:10.1186/s13287-018-0802-8 29482654 PMC5828113

[16] Zhou LN , Bi CS , Gao LN , An Y , Chen F , Chen FM . Macrophage polarization in human gingival tissue in response to periodontal disease. Oral Dis. 2019;25(1):265‐273. doi:10.1111/odi.12983 30285304

[17] He XT , Li X , Yin Y , Wu RX , Xu XY , Chen FM . The effects of conditioned media generated by polarized macrophages on the cellular behaviours of bone marrow mesenchymal stem cells. J Cell Mol Med. 2018;22(2):1302‐1315. doi:10.1111/jcmm.13431 29106032 PMC5783837

[18] Li X , He XT , Kong DQ , et al. M2 macrophages enhance the cementoblastic differentiation of periodontal ligament stem cells via the Akt and JNK pathways. Stem Cells. 2019;37(12):1567‐1580. doi:10.1002/stem.3076 31400241

[19] Medina CB , Mehrotra P , Arandjelovic S , et al. Metabolites released from apoptotic cells act as tissue messengers. Nature. 2020;580(7801):130‐135. doi:10.1038/s41586-020-2121-3 32238926 PMC7217709

[20] Liu X , Lieberman J . A mechanistic understanding of pyroptosis: the fiery death triggered by invasive infection. Adv Immunol. 2017;135:81‐117. doi:10.1016/bs.ai.2017.02.002 28826530 PMC10245508

[21] Man SM , Karki R , Kanneganti TD . Molecular mechanisms and functions of pyroptosis, inflammatory caspases and inflammasomes in infectious diseases. Immunol Rev. 2017;277(1):61‐75. doi:10.1111/imr.12534 28462526 PMC5416822

[22] Xia S , Hollingsworth LR , Wu H . Mechanism and regulation of gasdermin‐mediated cell death. Cold Spring Harb Perspect Biol. 2020;12(3):a036400. doi:10.1101/cshperspect.a036400 31451512 PMC7050592

[23] Jorgensen I , Miao EA . Pyroptotic cell death defends against intracellular pathogens. Immunol Rev. 2015;265(1):130‐142. doi:10.1111/imr.12287 25879289 PMC4400865

[24] Shi J , Gao W , Shao F . Pyroptosis: gasdermin‐mediated programmed necrotic cell death. Trends Biochem Sci. 2017;42(4):245‐254. doi:10.1016/j.tibs.2016.10.004 27932073

[25] Rao Z , Zhu Y , Yang P , et al. Pyroptosis in inflammatory diseases and cancer. Theranostics. 2022;12(9):4310‐4329. doi:10.7150/thno.71086 35673561 PMC9169370

[26] Zheng X , Chen W , Gong F , Chen Y , Chen E . The role and mechanism of pyroptosis and potential therapeutic targets in sepsis: a review. Front Immunol. 2021;12:711939. doi:10.3389/fimmu.2021.711939 34305952 PMC8293747

[27] Chen Q , Liu X , Wang D , et al. Periodontal inflammation‐triggered by periodontal ligament stem cell pyroptosis exacerbates periodontitis. Front Cell Dev Biol. 2021;9:663037. doi:10.3389/fcell.2021.663037 33869229 PMC8049442

[28] García‐Hernández AL , Muñoz‐Saavedra ÁE , González‐Alva P , et al. Upregulation of proteins of the NLRP3 inflammasome in patients with periodontitis and uncontrolled type 2 diabetes. Oral Dis. 2019;25(2):596‐608. doi:10.1111/odi.13003 30422379

[29] Yamaguchi Y , Kurita‐Ochiai T , Kobayashi R , Suzuki T , Ando T . Regulation of the NLRP3 inflammasome in porphyromonas gingivalis‐accelerated periodontal disease. Inflamm Res. 2017;66(1):59‐65. doi:10.1007/s00011-016-0992-4 27665233

[30] Li Y , Ling J , Jiang Q . Inflammasomes in alveolar bone loss. Front Immunol. 2021;12:691013. doi:10.3389/fimmu.2021.691013 34177950 PMC8221428

[31] Zhao P , Yue Z , Nie L , et al. Hyperglycaemia‐associated macrophage pyroptosis accelerates periodontal inflamm‐aging. J Clin Periodontol. 2021;48(10):1379‐1392. doi:10.1111/jcpe.13517 34219262

[32] Muendlein HI , Jetton D , Connolly WM , et al. cFLIP(L) protects macrophages from LPS‐induced pyroptosis via inhibition of complex II formation. Science. 2020;367(6484):1379‐1384. doi:10.1126/science.aay3878 32193329 PMC7375259

[33] Ma C , Yang D , Wang B , et al. Gasdermin D in macrophages restrains colitis by controlling cGAS‐mediated inflammation. Sci Adv. 2020;6(21):eaaz6717. doi:10.1126/sciadv.aaz6717 32671214 PMC7314554

[34] Shapouri‐Moghaddam A , Mohammadian S , Vazini H , et al. Macrophage plasticity, polarization, and function in health and disease. J Cell Physiol. 2018;233(9):6425‐6440. doi:10.1002/jcp.26429 PMC13482560 29319160

[35] Panigrahy D , Gilligan MM , Serhan CN , Kashfi K . Resolution of inflammation: an organizing principle in biology and medicine. Pharmacol Ther. 2021;227:107879. doi:10.1016/j.pharmthera.2021.107879 33915177

[36] Chen HW , Zhou W , Liao Y , Hu SC , Chen TL , Song ZC . Analysis of metabolic profiles of generalized aggressive periodontitis. J Periodontal Res. 2018;53(5):894‐901. doi:10.1111/jre.12579 29974463

[37] Kuboniwa M , Sakanaka A , Hashino E , Bamba T , Fukusaki E , Amano A . Prediction of periodontal inflammation via metabolic profiling of saliva. J Dent Res. 2016;95(12):1381‐1386. doi:10.1177/0022034516661142 27470067

[38] Caton JG , Armitage G , Berglundh T , et al. A new classification scheme for periodontal and peri‐implant diseases and conditions – Introduction and key changes from the 1999 classification. J Periodontol. 2018;89(Suppl 1):S1‐S8. doi:10.1002/jper.18-0157 29926946

[39] Tonetti MS , Greenwell H , Kornman KS . Staging and grading of periodontitis: framework and proposal of a new classification and case definition. J Periodontol. 2018;89(Suppl 1):S159‐S172. doi:10.1002/jper.18-0006 29926952

[40] Zhang J , An Y , Gao LN , Zhang YJ , Jin Y , Chen FM . The effect of aging on the pluripotential capacity and regenerative potential of human periodontal ligament stem cells. Biomaterials. 2012;33(29):6974‐6986. doi:10.1016/j.biomaterials.2012.06.032 22789721

[41] Dominici M , Le Blanc K , Mueller I , et al. Minimal criteria for defining multipotent mesenchymal stromal cells. The international society for cellular therapy position statement. Cytotherapy. 2006;8(4):315‐317. doi:10.1080/14653240600855905 16923606

[42] Viswanathan S , Shi Y , Galipeau J , et al. Mesenchymal stem versus stromal cells: international society for cell & gene therapy (ISCT®) mesenchymal stromal cell committee position statement on nomenclature. Cytotherapy. 2019;21(10):1019‐1024. doi:10.1016/j.jcyt.2019.08.002 31526643

[43] Zhang YL , Liu F , Li ZB , et al. Metformin combats high glucose‐induced damage to the osteogenic differentiation of human periodontal ligament stem cells via inhibition of the NPR3‐mediated MAPK pathway. Stem Cell Res Ther. 2022;13(1):305. doi:10.1186/s13287-022-02992-z 35841070 PMC9284897

[44] Zhou H , Li X , Yin Y , et al. The proangiogenic effects of extracellular vesicles secreted by dental pulp stem cells derived from periodontally compromised teeth. Stem Cell Res Ther. 2020;11(1):110. doi:10.1186/s13287-020-01614-w 32143712 PMC7060605

[45] Seidlitz EP , Sharma MK , Singh G . Extracellular glutamate alters mature osteoclast and osteoblast functions. Can J Physiol Pharmacol. 2010;88(9):929‐936. doi:10.1139/y10-070 20921979

[46] Takarada‐Iemata M , Takarada T , Nakamura Y , Nakatani E , Hori O , Yoneda Y . Glutamate preferentially suppresses osteoblastogenesis than adipogenesis through the cystine/glutamate antiporter in mesenchymal stem cells. J Cell Physiol. 2011;226(3):652‐665. doi:10.1002/jcp.22390 20717926

[47] Kesavardhana S , Malireddi RKS , Kanneganti TD . Caspases in cell death, inflammation, and pyroptosis. Annu Rev Immunol. 2020;38:567‐595. doi:10.1146/annurev-immunol-073119-095439 32017655 PMC7190443

[48] Mao CY , Wang YG , Zhang X , Zheng XY , Tang TT , Lu EY . Double‐edged‐sword effect of IL‐1β on the osteogenesis of periodontal ligament stem cells via crosstalk between the NF‐κB, MAPK and BMP/Smad signaling pathways. Cell Death Dis. 2016;7(7):e2296. doi:10.1038/cddis.2016.204 27415426 PMC4973347

[49] Sager HB , Heidt T , Hulsmans M , et al. Targeting interleukin‐1β reduces leukocyte production after acute myocardial infarction. Circulation. 2015;132(20):1880‐1890. doi:10.1161/circulationaha.115.016160 26358260 PMC4651795

[50] Vogel S , Börger V , Peters C , et al. Necrotic cell‐derived high mobility group box 1 attracts antigen‐presenting cells but inhibits hepatocyte growth factor‐mediated tropism of mesenchymal stem cells for apoptotic cell death. Cell Death Differ. 2015;22(7):1219‐1230. doi:10.1038/cdd.2014.225 25571972 PMC4572869

[51] Hajishengallis G , Korostoff JM . Revisiting the Page & Schroeder model: the good, the bad and the unknowns in the periodontal host response 40 years later. Periodontol 2000. 2017;75(1):116‐151. doi:10.1111/prd.12181 28758305 PMC5539911

[52] Fernandes‐Alnemri T , Wu J , Yu JW , et al. The pyroptosome: a supramolecular assembly of ASC dimers mediating inflammatory cell death via caspase‐1 activation. Cell Death Differ. 2007;14(9):1590‐1604. doi:10.1038/sj.cdd.4402194 17599095 PMC3345951

[53] Platnich JM , Chung H , Lau A , et al. Shiga toxin/lipopolysaccharide activates caspase‐4 and gasdermin d to trigger mitochondrial reactive oxygen species upstream of the NLRP3 inflammasome. Cell Rep. 2018;25(6):1525‐1536.e7. doi:10.1016/j.celrep.2018.09.071 30404007

[54] Robinson N , Ganesan R , Hegedűs C , Kovács K , Kufer TA , Virág L . Programmed necrotic cell death of macrophages: focus on pyroptosis, necroptosis, and parthanatos. Redox Biol. 2019;26:101239. doi:10.1016/j.redox.2019.101239 31212216 PMC6582207

[55] He WT , Wan H , Hu L , et al. Gasdermin D is an executor of pyroptosis and required for interleukin‐1β secretion. Cell Res. 2015;25(12):1285‐1298. doi:10.1038/cr.2015.139 26611636 PMC4670995

[56] Wu D , Zhu X , Ao J , Song E , Song Y . Delivery of ultrasmall nanoparticles to the cytosolic compartment of pyroptotic J774A.1 macrophages via GSDMD(Nterm) membrane pores. ACS Appl Mater Interfaces. 2021;13(43):50823‐50835. doi:10.1021/acsami.1c17382 34689556

[57] Frank D , Vince JE . Pyroptosis versus necroptosis: similarities, differences, and crosstalk. Cell Death Differ. 2019;26(1):99‐114. doi:10.1038/s41418-018-0212-6 30341423 PMC6294779

[58] Jiao Y , Zhang T , Zhang C , et al. Exosomal miR‐30d‐5p of neutrophils induces M1 macrophage polarization and primes macrophage pyroptosis in sepsis‐related acute lung injury. Crit Care. 2021;25(1):356. doi:10.1186/s13054-021-03775-3 34641966 PMC8507252

[59] Li N , Chen J , Geng C , et al. Myoglobin promotes macrophage polarization to M1 type and pyroptosis via the RIG‐I/Caspase1/GSDMD signaling pathway in CS‐AKI. Cell Death Discov. 2022;8(1):90. doi:10.1038/s41420-022-00894-w 35228524 PMC8885737

[60] Sica A , Mantovani A . Macrophage plasticity and polarization: in vivo veritas. J Clin Invest. 2012;122(3):787‐795. doi:10.1172/jci59643 22378047 PMC3287223

[61] Ben‐Sasson SZ , Hogg A , Hu‐Li J , et al. IL‐1 enhances expansion, effector function, tissue localization, and memory response of antigen‐specific CD8 T cells. J Exp Med. 2013;210(3):491‐502. doi:10.1084/jem.20122006 23460726 PMC3600912

[62] Du T , Gao J , Li P , et al. Pyroptosis, metabolism, and tumor immune microenvironment. Clin Transl Med. 2021;11(8):e492. doi:10.1002/ctm2.492 34459122 PMC8329701

