# Supplementary material for: Pyroptotic macrophages induce disruption of glutamate metabolism in periodontal ligament stem cells contributing to their compromised osteogenic potential
Source: Cell Prolif. 2024 May 27;57(10):e13663. doi: 10.1111/cpr.13663 (PMC11471398; doi:10.1111/cpr.13663)
Supplement: Supplementary file 1 — Data S1: Supporting Information. [file CPR-57-e13663-s001.docx]

**Additional file 1:** **Supplementary materials**

**Pyroptotic macrophages induce disruption of glutamate metabolism in periodontal ligament stem cells contributing to their compromised osteogenic potential**

Li-Juan Sun, Hong-Lei Qu, Xiao-Tao He, Bei-Min Tian, Rui-Xin Wu, Yuan Yin, Jie-Kang Zou, Hai-Hua Sun, Xuan Li, Fa-Ming Chen

**Additional file 1 Includes the Following:**

**Supplementary Figure (3)**

**Supplementary Tables (3)**


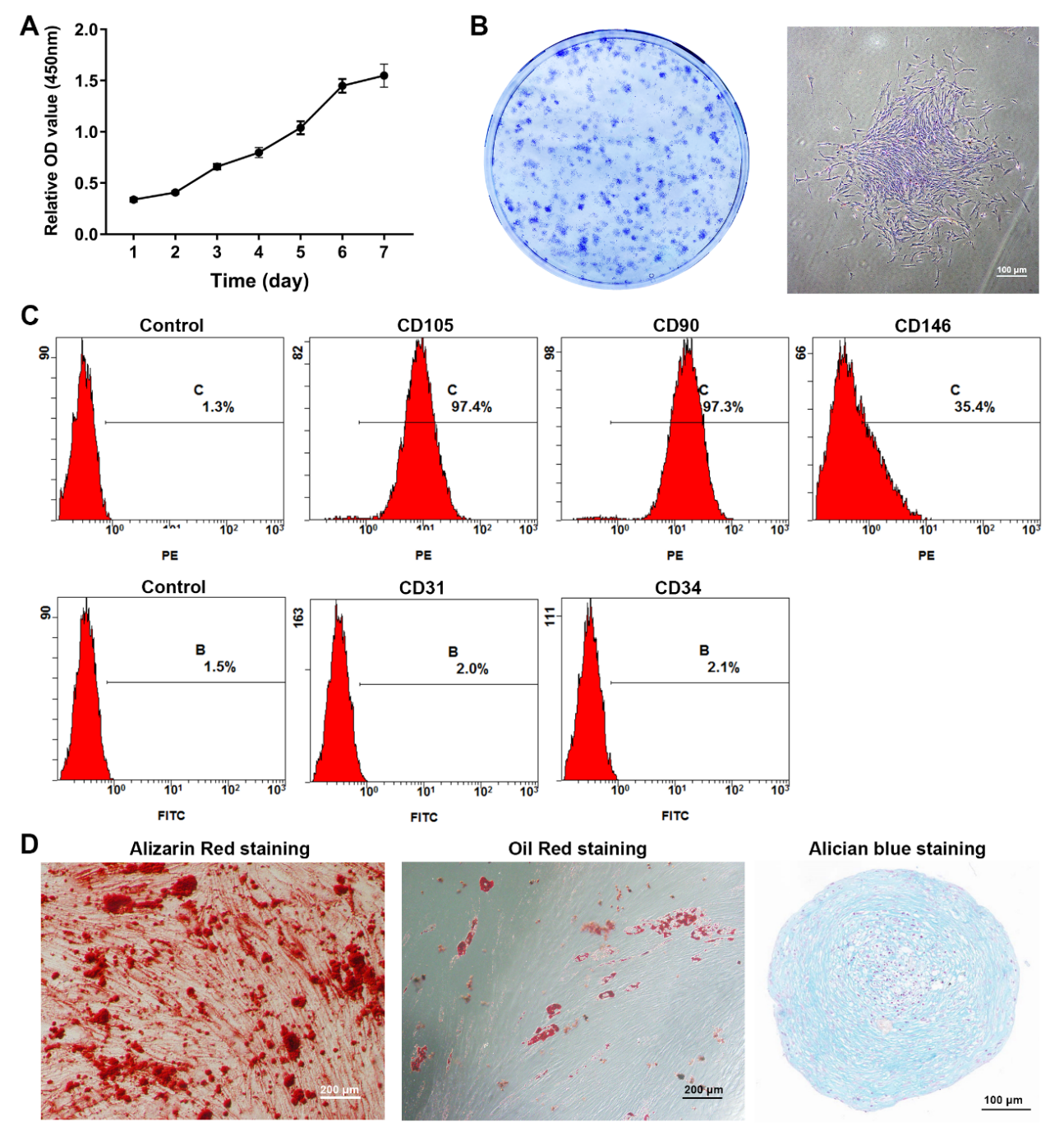


**Fig. S1.** Identification of human PDLSCs. (A) Growth curve of PDLSCs determined by CCK-8 assay. (B) Representative images of PDLSC colonies in the dish and a single colony amplified by microscope (scale bar: 100 μm). (C) Flow cytometric analysis of PDLSC surface markers. (D) Osteogenic (Alizarin Red S staining, left, scale bar: 200 μm), adipogenic (Oil Red O staining, middle, scale bar: 200 μm) and chondrogenic (Alcian blue staining, right, scale bar: 100 μm) differentiation assays of PDLSCs in vitro.


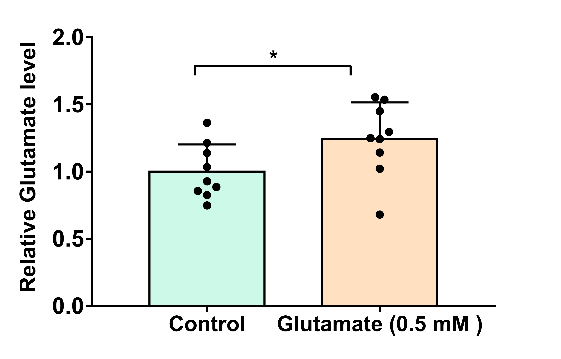


**Fig. S2.** Exogenous glutamate (0.5 mM) increased the intracellular glutamate level of CM-treated PDLSCs. Data are shown as mean ± SD and analyzed by t test. Significant differences between two groups are represented by * (*P* < 0.05).


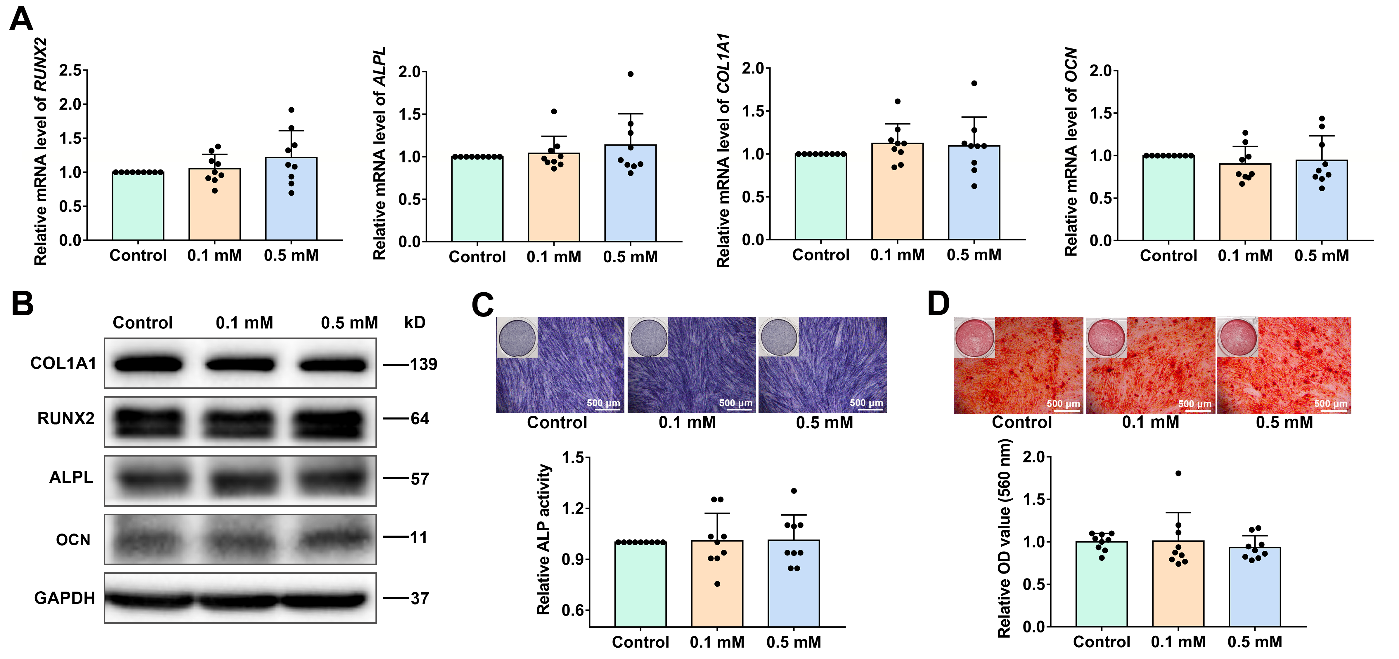


**Fig. S3.** Exogenous glutamate had no effect on the osteogenic differentiation of PDLSCs. PDLSCs cultured in normal medium were divided into three groups, two of which were supplemented with 0.1mM and 0.5mM glutamate separately, with the remaining group served as control group. (A) Relative expressions of osteogenic differentiation-related genes *COL1A1*, *ALPL*, *RUNX2* and *OCN* analyzed by qRT-PCR. (B) Western blot for osteogenic differentiation-related protein levels of COL1A1, ALPL, RUNX2 and OCN. (C) ALP staining (scale bar: 500 µm) and cellular ALP activity assays. (D) Alizarin Red staining (scale bar: 500 µm) and quantitative assays. Data are shown as mean ± SD and analyzed by one-way ANOVA. There is no significant difference between groups.

**Supplementary Tables**

**Table S1.** Sequences of gene-specifc primers for qRT-PCR

| **Gene** | **Primer** | **Sequences (5’-3’)** |
| --- | --- | --- |
| *GAPDH* | Forward | GGAGTCCACTGGCGTCTTCA |
|  | Reverse | GTCATGAGTCCTTCCACGATACC |
| *ALPL* | Forward | AACATCAGGGACATTGACGTG |
|  | Reverse | GTATCTCGGTTTGAAGCTCTTCC |
| *RUNX2* | Forward | TGGTTACTGTCATGGCGGGTA |
|  | Reverse | TCTCAGATCGTTGAACCTTGCTA |
| *COL1A1* | Forward | GAGGGCCAAGACGAAGACATC |
|  | Reverse | CAGATCACGTCATCGCACAAC |
| *OCN* | Forward | CCCAGGCGCTACCTGTATCAA |
|  | Reverse | GGTCAGCCAACTCGTCACAGTC |
| *SLC7A11* | Forward | TCTCCAAAGGAGGTTACCTGC |
|  | Reverse | AGACTCCCCTCAGTAAAGTGAC |
| *GCLC* | Forward | AGTTGAGGCCAACATGCGAA |
|  | Reverse | CATCTCCACCAACACAGACA |
| *GCLM* | Forward | TGTCTTGGAATGCACTGTATCTC |
|  | Reverse | CCCAGTAAGGCTGTAAATGCTC |
| *GSS* | Forward | AGCTTTCCATCTGAGGACCAG |
|  | Reverse | TCCTATCCCAAGTCAGGCACT |

**Table S2.** List of the top 10 regulated metabolites in CM-treated PDLSCs compared with those in PDLSCs

| **Metabolite** | **Class** | **Regulated** | ***p*-value** | **VIP** | **FC** |
| --- | --- | --- | --- | --- | --- |
| Dihydromacarpine | - | down | 0.006734 | 1.293086 | 0.019167 |
| S-Lactoylglutathione | Carboxylic acids and derivatives | down | 0.000228 | 1.068812 | 0.031908 |
| D-Urobilin | - | down | 0.005081 | 1.285539 | 0.037 |
| O-Succinyl-L-homoserine | - | down | 0.005195 | 1.057031 | 0.046634 |
| Gluconic Acid | Organooxygen compounds | down | 0.00023 | 1.107173 | 0.04983 |
| 3'-CMP | Organooxygen compounds | up | 2.18E-05 | 1.239611 | 16.05389 |
| Glutathione | Carboxylic acids and derivatives | down | 0.000152 | 1.368746 | 0.06469 |
| Lotaustralin | Organooxygen compounds | down | 0.000224 | 1.223505 | 0.068125 |
| Methylimidazole acetaldehyde | Azoles | down | 0.005054 | 1.271396 | 0.070648 |
| Jadomycin A | - | down | 7.00E-05 | 1.062227 | 0.075217 |

**Table S3.** The top ten central nodes in DEM network analysis

| **Id** | **Label** | **Degree** | **Betweenness** |
| --- | --- | --- | --- |
| C00025 | L-Glutamic Acid | 294 | 86859.41 |
| C00021 | S-Adenosylhomocysteine | 176 | 46527.91 |
| C00158 | Citric Acid | 136 | 25822.58 |
| C00547 | Norepinephrine | 105 | 16331.72 |
| C00073 | L-Methionine | 96 | 9942.28 |
| C00788 | Epinephrine | 93 | 7638.36 |
| C00051 | Glutathione | 91 | 12924 |
| C00334 | Gamma-Aminobutyric Acid | 85 | 7884.83 |
| C00079 | L-Phenylalanine | 79 | 6466.86 |
| C00099 | Beta-Alanine | 75 | 7883.97 |
